# Effects of platelet-rich plasma gel on vaginal microecology and stump healing and its underlying mechanism

**DOI:** 10.3389/fmed.2025.1743988

**Published:** 2026-01-02

**Authors:** Mingyang Wang, Yunbi Peng, Li Zhou, Hong Liu, Xiaofeng Zou, Zhiliang Wang

**Affiliations:** 1 Department of Obstetrics and Gynecology, Affiliated Hospital of Zunyi Medical University, Zunyi, Guizhou, China; 2 Department of Obstetrics and Gynecology, Kweichow Moutai Hospital, Zunyi, Guizhou, China

**Keywords:** lactobacillus, vaginal stump, vaginal microecology, inflammatory factors, TLR4, MBL, laparoscopic hysterectomy

## Abstract

**Objective:**

The study aimed to explore the effects of platelet-rich plasma (PRP) gel on vaginal microecology and stump healing, as well as its underlying mechanism.

**Methods:**

From December 2022 to August 2024, 100 patients who underwent laparoscopic hysterectomy at our hospital were selected and divided into an observation group and a control group. The control group received the conventional suture method, while the observation group received PRP glue applied between the reflexive peritoneum and the vaginal stump in addition to the conventional suture.

**Results:**

Compared to the control group, the observation group had a higher rate of grade A healing (*p* < 0.05) and a lower rate of bleeding and granulation in the vaginal stump (*p* < 0.05). One month after surgery, compared to the control group, the observation group had a greater number of patients with cleanliness levels I-II, a smaller number of patients with cleanliness level IV, a smaller number of patients with pH values > 4.5, and lower positive rates of sialic acid enzyme activity, hydrogen peroxide concentration, and leukocyte esterase activity (*p* < 0.05). The total pathogen infection rate in the observation group was lower than that in the control group (*p* < 0.05). Furthermore, 3 days after surgery, compared to the control group, the observation group had lower levels of CRP, IL-6, IL-11, IL-21, and IL-2, as well as lower levels of toll-like receptor 4 (TLR4) and MBL (*p* < 0.05).

**Conclusion:**

PRP gel can improve vaginal microecology and promote the healing of the vaginal stump in patients undergoing laparoscopic hysterectomy. However, further research is needed to fully understand the underlying mechanisms and confirm its long-term effects.

## Introduction

The incidence of adenomyosis and hysteromyoma has been rising in recent years, leading to increased use of laparoscopic hysterectomy (1). However, this surgical procedure can lead to several issues that affect vaginal health. The use of antibiotics before and after surgery can disrupt the normal vaginal flora (2). Moreover, after a hysterectomy, the vaginal microecological anatomy is disrupted, as the vagina is no longer intact. In addition, long-term overflow of ascites through the vaginal stump neutralizes the natural acidic environment of the vagina, causing serious damage to its microecology (3).

Platelet-rich plasma (PRP) is a concentrate of platelets and bioactive factors derived from the concentration and processing of human blood (4). These bioactive factors, including growth factors such as platelet-derived growth factor (PDGF), transforming growth factor beta (TGF-*β*), and vascular endothelial growth factor (VEGF), play crucial roles in tissue repair and regeneration (5). They can stimulate cell proliferation, migration, and differentiation, as well as promote angiogenesis and extracellular matrix synthesis (6).

In the field of gynecology, PRP has shown potential in promoting the healing of various tissues (7–9). For example, in pelvic floor reconstruction, PRP enhances the repair of damaged tissues by promoting fibroblast proliferation and collagen synthesis, thereby improving the structural integrity and function of the pelvic floor (10). Another study on endometrial repair after curettage or other uterine procedures found that PRP can stimulate endometrial cell proliferation and angiogenesis, promoting faster and more complete recovery of the endometrium (11). Given these properties of PRP, it is reasonable to hypothesize that it could also have a positive effect on the healing of the vaginal stump. The vaginal stump, after hysterectomy, exists in a disrupted microecological environment and requires effective repair mechanisms. The bioactive factors in PRP may help in this process by promoting cell-related activities and tissue regeneration.

In our study, we aimed to explore the effects of PRP gel on vaginal microecology and its role in promoting the healing of the vaginal stump, as well as its underlying mechanism.

## Data and methods

### Study design

A total of 100 patients who underwent laparoscopic hysterectomy at our hospital from December 2022 to August 2024 were selected as study participants. This study was approved by the Ethics Committee of the Affiliated Hospital of Zunyi Medical University. All patients signed the informed consent form.

The inclusion criteria were as follows: (1) Patients who met the diagnostic criteria for relevant obstetric and gynecological diseases, as outlined in standard clinical guidelines; (2) no history of sex hormone use, such as contraceptive drugs, within the past month; (3) no antibiotic use within the past week; (4) no history of glucocorticoid use within the past 3 months; and (5) no other ongoing infections at the time of enrollment.

The exclusion criteria were as follows: (1) Patients with known immune-related diseases; (2) patients with abnormal liver and kidney function; (3) patients diagnosed with diabetes mellitus; (4) patients with active reproductive tract infections; (5) patients with malignant tumors; and (6) patients who had engaged in sexual intercourse within the 3 days prior to the study.

### Randomization

The patients were randomly divided into an observation group and a control group. The randomization process was carried out using the random number table method. First, a random number table was generated. Then, each patient was assigned a unique sequential number from 1 to 100. According to the order of these numbers, the patients were matched with the numbers in the random number table. Starting from a randomly chosen position in the table, the numbers were read sequentially. The patients were divided into the observation group and the control group, with 50 cases in each group. The patients corresponding to odd-numbered random numbers were allocated to the observation group, while those corresponding to even-numbered random numbers were allocated to the control group.

### Blinding

This study employed a double-blind design. The patients were unaware of whether they were in the observation group or the control group to avoid potential psychological influences on the study results. Similarly, the researchers who were responsible for evaluating outcomes, such as the healing of the vaginal stump, were also blinded to the group allocation of the patients. This was achieved by having an independent researcher who was not involved in the outcome assessment perform the group allocation and keep the group information confidential until the end of the study and data analysis.

### Sample size calculation

We assumed an anticipated effect size (Cohen’s *d*) of 0.8, representing a relatively large and clinically meaningful difference between the observation group and the control group in terms of vaginal stump healing (12). With a significance level (*α*) of 0.05 (two-tailed test) and a desired power (1-*β*) of 0.8, the estimated sample size for each group was approximately 50, as calculated using G*Power.

## Methods

### Preparation of PRP gel

A total of 20 mL of venous blood was collected from each patient using an EDTA-sterile vacuum anticoagulant tube. The extracted blood was promptly transferred to glass centrifuge tubes, with each tube containing 10 mL. The tubes were placed symmetrically in the centrifuge to ensure balanced rotation. The centrifuge was then started, and the required PRP products were generated through a one-step centrifugation process. The centrifugation process was performed at 2,000 rpm for 10 min. After centrifugation, the blood in the tubes separated into different layers, with the PRP layer located between the upper plasma layer and the lower red blood cell layer. A toothed forceps was used to carefully obtain the gel-like portion of the PRP. The underlying blood components, primarily red blood cells and some plasma, were removed. The resulting pale-yellow substance was the PRP gel. The platelet concentration in the prepared PRP gel was measured using an automated hematology analyzer. On average, the platelet concentration in the PRP gel was three times higher than that in whole blood. To ensure standardization, all samples were processed in the same laboratory under identical environmental conditions, and the same batch of reagents and equipment was used throughout the study. This minimized any potential variations in the platelet concentration measurement and ensured the consistency and reproducibility of the results. For activation, calcium gluconate was added to the PRP gel at a ratio of 1:10. The mixture was then gently agitated and allowed to stand for 5 min to form a stable gel structure for subsequent use in the study.

### Surgical methods

Control group: 1 day before surgery, the umbilical cord was cleaned in the evening as part of routine preparation, and alcohol consumption and fasting were prohibited for 10 h. The vulvar skin was prepared, the patient was placed in the lithotomy position, tracheal intubation and general anesthesia were administered, and a urinary catheter was inserted. An incision of approximately 1 cm in length was made in the umbilical cord, and the skin tissue was dissected layer by layer down to the peritoneum. A 10 mm puncture device was used and vertically inserted into the incision, and aeration treatment was carried out. The pneumoperitoneum pressure was maintained at 14 mmHg with CO_2_ insufflation. The patient was placed in a 30° Trendelenburg position (head-down, foot-up), and a laparoscope was introduced through the umbilical incision. Under laparoscopic guidance, two additional 5-mm trocars were inserted at the McBurney point and its contralateral mirror point to allow comprehensive exploration of the abdominal cavity. The bilateral round ligaments and ovarian ligaments were then coagulated. The broad ligaments of the uterus were cut and coagulated toward the cervix up to the outer edge of the uterine isthmus, and hysterectomy was performed with laparoscopic assistance. The uterine cup was placed in the vagina, the bladder was mobilized and returned to the peritoneal cavity, the hysterectomy was completed using an electrosurgical hook, and the vaginal stump was sutured with standard 1–0 absorbable sutures.

Based on the procedure used in the control group, PRP gel was applied between the reflected peritoneum and the vaginal stump in the observation group.

### Observation indicators

(1) The healing of the vaginal stump was assessed and classified into three distinct levels: Grade A healing: This level represents the most favorable healing outcome. The incision of the vaginal stump demonstrates complete and optimal healing. There is an absence of hyperemia, which means the surrounding tissue shows no abnormal redness or increased blood flow, indicating a lack of inflammation. The wound edges are neatly approximated, and the overall appearance of the healing site is smooth and healthy. In addition, there is no evidence of any fluid leakage or abnormal discharge. The patient typically experiences minimal to no discomfort at the surgical site, and normal physiological functions in the vaginal area are gradually restored. Grade B healing: Grade B healing is characterized by sub-optimal healing conditions. The healing process is not as smooth as in Grade A. There are visible defects at the incision site. The broken end of the vaginal stump exhibits signs of inflammation, such as congestion, manifested by localized redness and swelling due to increased blood accumulation. However, it is crucial to note that there is no suppuration, which means no pus formation is present. The wound edges may not be as closely aligned as in Grade A healing, and the overall healing progress is slower. Patients with Grade B healing may experience mild to moderate discomfort, including a sense of fullness or slight pain in the vaginal region. Grade C healing: This represents the most severe level of healing impairment. The incision of the vaginal stump becomes infected, as evidenced by the presence of purulent discharge. The infection can lead to significant redness, swelling, and warmth in the surrounding tissue. The wound edges are often separated, creating an open area that is prone to further bacterial invasion. In addition to assessing the healing of the vaginal stump itself, we also closely monitored other related signs. Congestion and bleeding at the vaginal stump site were carefully noted. Congestion, as mentioned earlier, indicates increased blood flow and potential inflammation, while bleeding can range from minor spotting to more significant hemorrhage, which may require medical intervention. Granulation tissue formation was also evaluated. Granulation tissue is a normal part of the wound-healing process, but excessive or abnormal granulation can be a sign of chronic inflammation or improper healing. In Grade C healing, the granulation tissue may be overgrown, irregular in shape, or easily bleed upon touch.
(2) One month after surgery, vaginal microecology was assessed, including measurements of cleanliness, pH, sialidase activity, hydrogen peroxide concentration, and leukocyte esterase activity.

The cleanliness of the vaginal environment was evaluated on a scale from I to IV. A cleanliness degree of III or IV was considered abnormal. Degree I: The vaginal secretion is clear or translucent, with a small amount of mucus. There is no obvious odor, and microscopic examination reveals few or no white blood cells (less than 5 per high-power field). This indicates healthy and well-balanced vaginal microecology. Degree II: The vaginal secretion is slightly turbid, with a moderate amount of mucus and a mild odor. Microscopic examination reveals a small number of white blood cells (5–15 per high-power field). This may suggest a slight imbalance in vaginal microecology, but it is generally not associated with significant infection. Degree III: The vaginal secretion is turbid, with a large amount of pus-like or mucopurulent discharge and a strong odor. Microscopic examination shows a large number of white blood cells (15–30 per high-power field). This is often indicative of a vaginal infection, such as bacterial vaginosis or a sexually transmitted infection. Degree IV: The vaginal secretion is purulent, with a very strong odor, and microscopic examination reveals a very high number of white blood cells (more than 30 per high-power field). This represents a severe vaginal infection that requires immediate medical attention.

pH detection: The normal pH value of the vaginal environment ranges from 3.8 to 4.5. A pH value greater than 4.5 was considered abnormal. An elevated pH value can disrupt the normal acidic environment of the vagina, which is crucial for maintaining the growth of beneficial lactobacilli and inhibiting the growth of harmful pathogens. A pH > 4.5 may indicate a reduction in the number of lactobacilli and an increased risk of infection, such as bacterial vaginosis.

Sialidase activity detection: A positive sialidase activity test was used as a diagnostic marker for bacterial vaginosis. The presence of sialidase indicates bacterial overgrowth and a disruption of the normal vaginal flora balance.

Hydrogen peroxide concentration detection: A positive result for hydrogen peroxide concentration detection suggests the presence of a sufficient number of lactobacilli, which help maintain a healthy vaginal environment by producing antimicrobial substances and lowering the pH value. A negative result may indicate a reduction in the number of lactobacilli and an increased risk of infection. In our study, a positive hydrogen peroxide result was considered part of the criteria for normal vaginal flora, while a negative result, along with other abnormal findings, could contribute to the diagnosis of vaginal flora disorders.

Leukocyte esterase activity detection: A positive leukocyte esterase activity test indicates the presence of an inflammatory response in the vaginal tract, often associated with an infection. White blood cells are part of the body’s immune system, and their presence in vaginal secretions, as indicated by a positive leukocyte esterase test, suggests that the body is fighting an infection. In our scoring system, a positive leukocyte esterase result, along with other abnormal parameters, was considered indicative of vaginal flora disorders.

(3) A total of 5 mL of fasting venous blood was obtained from the patients, and serum was separated. Interleukin (IL)-6, IL-11, IL-21, and IL-2 levels were examined using ELISA kits before and 3 days after surgery.
(4) A total of 5 mL of fasting venous blood was obtained from the patients, and serum was separated. MBL and toll-like receptor 4 (TLR4) levels were examined using ELISA kits before and 3 days after surgery.

### Statistical analysis

Statistical analysis was performed using SPSS 19.0. For categorical variables, which were presented as frequencies and percentages [*n* (%)], the *χ*^2^ test was used to compare differences between the two groups. For continuous variables, which were presented as the mean ± standard deviation (x̅±s), the Shapiro–Wilk test was first used to assess the normality of the data distribution within each group. If the data in both groups followed a normal distribution, an independent samples *t*-test was performed. If the data in both groups did not follow a normal distribution, the Mann–Whitney *U* test was performed to compare differences between the two groups. Multiple comparisons between the different groups were analyzed using two-way ANOVA, and 95% confidence intervals (95% CI) were reported. *Post-hoc* analysis was performed using the Bonferroni–Dunn method to adjust for multiple comparisons. A *p*-value of <0.05 was considered statistically significant.

## Results

### General data of the patients in the two groups

The patients in the observation group were aged 38–70 years, and the mean age was 43.95 ± 5.62 years. Among them, 13 had ovarian cysts, 18 had cervical lesions (cervical intraepithelial lesion grade II-III), six had uterine adenomyosis, and 13 had uterine fibroids. In addition, there were 30 patients with human papillomavirus (HPV) infection. The patients in the control group were aged 37–68 years, and the mean age was (44.06 ± 5.67) years. Among them, 14 had ovarian cysts, 17 cervical lesions (cervical intraepithelial lesion grade II-III), seven had uterine adenomyosis, and 12 had uterine fibroids. In addition, there were 32 patients with HPV infection. No differences were observed in the general characteristics between the two groups (*p* > 0.05), indicating that the groups were comparable.

### Healing of the vaginal stump between the two groups

The rate of grade A healing in the observation group was higher than that in the control group (*p* = 0.029, 95% CI: 0.447–0.953), while the rate of bleeding and granulation in the vaginal stump in the observation group was lower than that in the control group (*p* = 0.047, 95% CI: 1.005–2.157), as shown in Table 1. These results suggest that PRP gel can promote the healing of the vaginal stump in patients undergoing laparoscopic hysterectomy.

**Table 1:** Healing of the vaginal stump in the two groups [*n* (%)].

Groups	*n*	Grade A healing	Grade B healing	Grade C healing	Bleeding in the vaginal stump	Granulation in the vaginal stump
Control group	50	30 (60.00)	19 (38.00)	1 (2.00)	7 (14.00)	8 (16.00)
Observation group	50	40 (80.00)	10 (20.00)	0 (0.00)	1 (2.00)	1 (2.00)
*χ* ^2^		4.762	3.934	2.444	4.891	5.983
*p*		0.029	0.047	0.118	0.027	0.014

### Vaginal microecology between the two groups

One month after surgery, the proportion of patients with cleanliness degree I to II in the observation group was higher than that in the control group (*p* = 0.001, 95% CI: 0.344–0.777). The proportion of patients with cleanliness degree IV in the observation group was lower than that in the control group (*p* = 0.014, 95% CI: 1.181–2.550). The number of patients with pH > 4.5 in the observation group was lower than that in the control group (*p* = 0.022, 95% CI: 1.068–2.300). In the control group, the sialidase activity test was positive in eight patients, the hydrogen peroxide concentration test was positive in 20 patients, and the leukocyte esterase activity test was positive in 26 patients. In the observation group, the sialidase activity test was positive in one patient, the hydrogen peroxide concentration test was positive in seven patients, and the leukocyte esterase activity test was positive in 11 patients. Compared to the control group, the observation group had lower positive rates of sialidase activity, hydrogen peroxide concentration, and leucocyte esterase activity (*p* = 0.014, 95% CI: 1.181–2.550; *p* = 0.003, 95% CI: 1.235–2.558; *p* = 0.001, 95% CI: 1.262–2.713). In the control group, four patients were infected with *Trichomonas vaginalis*, three patients were infected with *Candida albicans*, and six patients were infected with mixed bacteria. The total infection rate was 26.00%. In the observation group, one patient was infected with *Candida albicans* and two patients were infected with mixed bacteria. The total infection rate was 6.00%. The total pathogen infection rate in the observation group was lower than that in the control group (*p* = 0.006, 95% CI: 1.224–2.526), as shown in Table 2. These results suggest that PRP gel has a positive effect on improving the vaginal microecology of patients undergoing laparoscopic hysterectomy.

**Table 2:** Vaginal microecology in the two groups [*n* (%)].

Groups	*n*	Cleanliness (degree)			pH > 4.5	Positive sialidase activity	Positive hydrogen peroxide concentration	Positive leukocyte esterase activity	Pathogen			
		I-II	III	IV					*Trichomonas vaginalis*	*Candida albicans*	Mixed bacteria	Total
Control group	50	20 (40.00)	22 (44.00)	8 (16.00)	24 (48.00)	8 (16.00)	20 (40.00)	26 (52.00)	4 (8.00)	3 (6.00)	6 (12.00)	13 (26.00)
Observation group	50	36 (72.00)	13 (26.00)	1 (2.00)	13 (26.00)	1 (2.00)	7 (14.00)	11 (22.00)	0 (0.00)	1 (2.00)	2 (4.00)	3 (6.00)
*χ* ^2^		10.39	3.560	5.983	5.191	5.983	8.574	9.653	7.440			
*p*		0.001	0.059	0.014	0.022	0.014	0.003	0.001	0.006			

### Levels of inflammatory factors between the two groups

**Figure 1:**
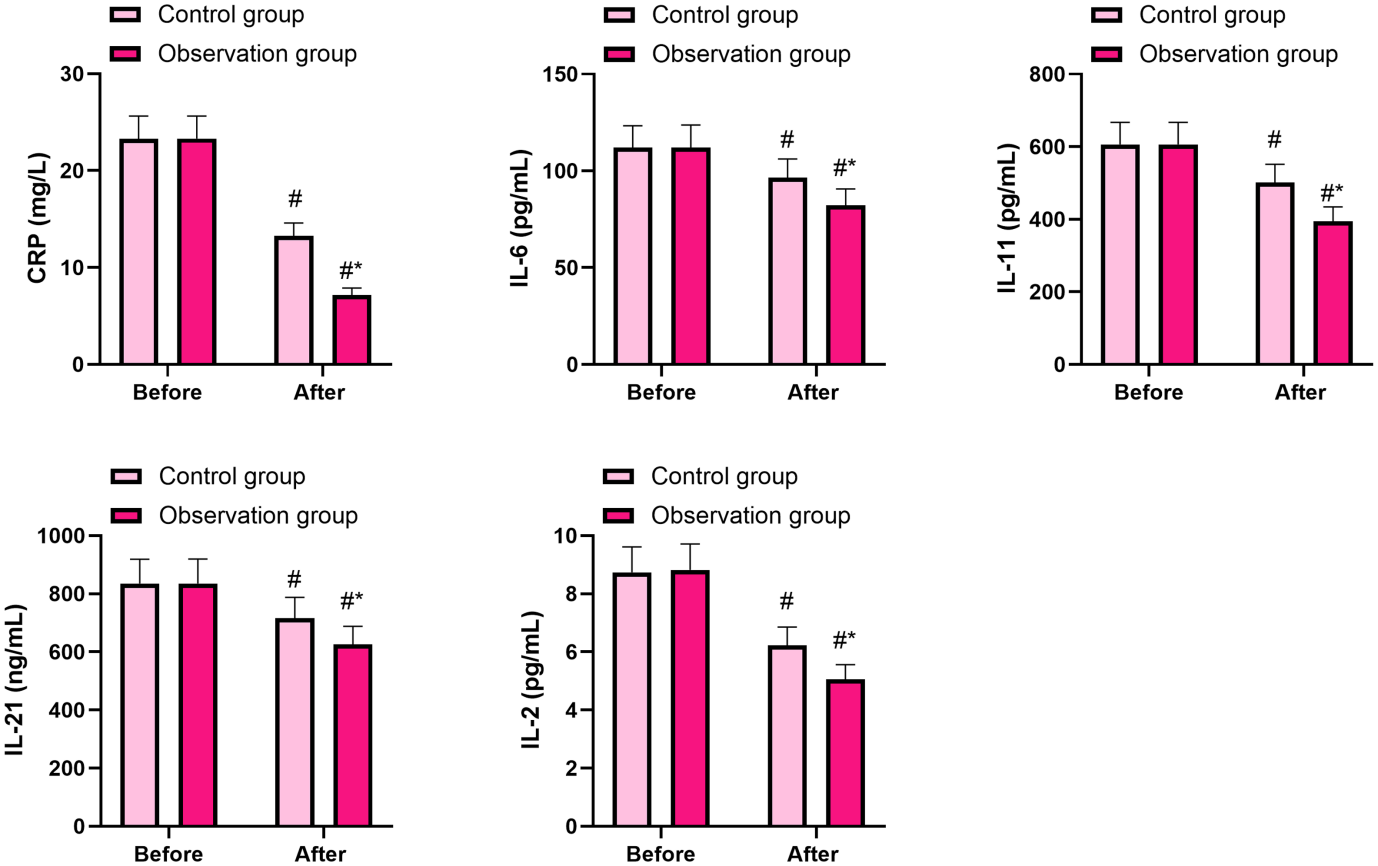
Comparison of the levels of inflammatory factors between the two groups. The data were presented as mean ± standard deviation. The symbol “#” indicates a statistically significant difference (*p* < 0.05) compared to the pre-treatment levels within the same group. The symbol “*” denotes a statistically significant difference (*p* < 0.05) between the observation group and the control group at the same time point.

Before therapy, no difference was observed in the levels of CRP, IL-6, IL-11, IL-21, and IL-2 between the two groups (*p* > 0.05). Three days after therapy, the levels of CRP, IL-6, IL-11, IL-21, and IL-2 decreased in both groups (*p* < 0.05, 95% CI: 2.523–3.537; *p* < 0.05, 95% CI: 4.214–9.956; *p* < 0.05, 95% CI: 38.55–68.44; *p* < 0.05, 95% CI: 24.26–66.59; *p* < 0.05, 95% CI: 0.343–0.756), and their levels in the observation group were lower than those in the control group (*p* < 0.05, 95% CI: 12.58–13.60; *p* < 0.05, 95% CI: 19.77–25.52; *p* < 0.05, 95% CI: 143.7–173.6; *p* < 0.05, 95% CI: 143.5–185.8; *p* < 0.05, 95% CI: 2.933–3.347), as shown in Figure 1. These results suggest that PRP gel can inhibit the inflammatory response of patients undergoing laparoscopic hysterectomy.

### Levels of TLR4 and MBL between the two groups

**Figure 2:**
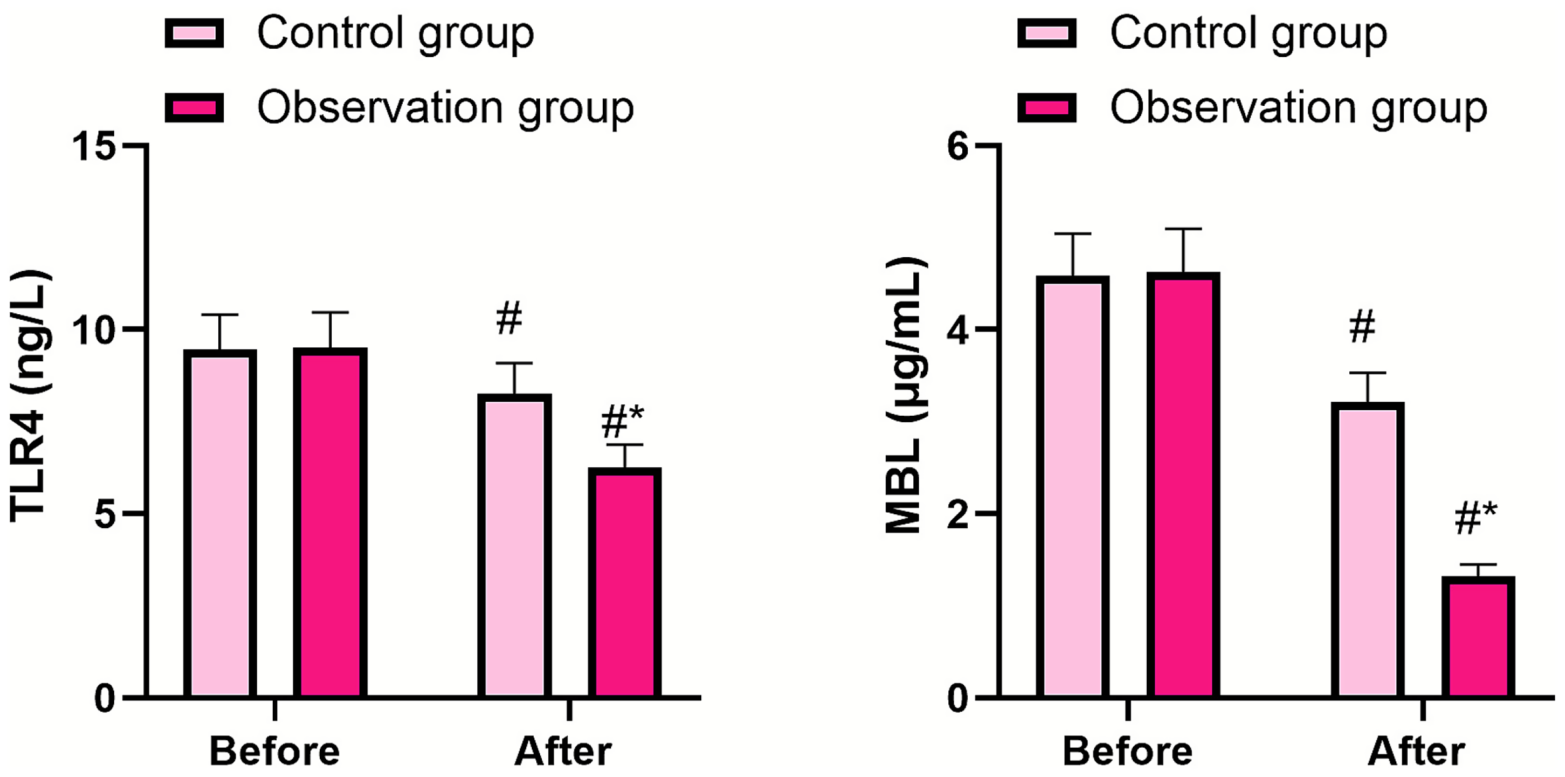
Comparison of the levels of IL-2, TLR4, and MBL between the two groups. The data were presented as mean ± standard deviation. The symbol “#” indicates a statistically significant difference (*p* < 0.05) compared to the pre-treatment levels within the same group. The symbol “*” denotes a statistically significant difference (*p* < 0.05) between the observation group and the control group at the same time point.

Before therapy, no difference was observed in TLR4 and MBL levels between the two groups (*p* > 0.05). Three days after therapy, TLR4 and MBL levels decreased in both groups (*p* < 0.05, 95% CI: 0.742–1.218; *p* < 0.05, 95% CI: 0.820–1.029), and their levels in the observation group were lower than those in the control group (*p* < 0.05, 95% CI: 1.992–2.468; *p* < 0.05, 95% CI: 2.231–2.439), as shown in Figure 2. These results suggest that PRP gel can inhibit the levels of TLR4 and MBL in patients undergoing laparoscopic hysterectomy.

## Discussion

Our study demonstrated that PRP gel has a positive impact on improving vaginal microecology and promoting the healing of the vaginal stump in patients undergoing laparoscopic hysterectomy. In the observation group, the rate of grade A healing was higher, while the rates of bleeding and granulation in the vaginal stump were lower compared to the control group. One month after surgery, a higher proportion of patients in the observation group had a vaginal cleanliness degree of I-II, while lower proportions of patients had a cleanliness degree of IV, a vaginal pH > 4.5, positive sialidase activity, positive hydrogen peroxide concentration, and positive leucocyte esterase activity. In addition, the total pathogen infection rate in the observation group was lower. These results suggest that PRP gel can effectively enhance the healing process and improve the vaginal microenvironment. Our findings are consistent with previous research in the context of endometrial repair and ovarian rejuvenation. For example, Qi et al. (13) demonstrated the use of locationally activated PRP delivered via an injectable dual-network hydrogel for endometrial regeneration. Wang et al. (14) suggested that PRP promotes the repair and regeneration of damaged endometrium in rats. Park et al. (15) proposed that using PRP for ovarian regeneration offers a highly promising option for women in the early menopausal stage to restore fertility. Garavelas et al. (16) suggested that intraovarian infusion of autologous PRP exhibits promising results for restoring ovarian insufficiency. Similar to these findings, our study suggests that PRP gel may promote tissue repair and regeneration and modulate the local tissue environment in the vaginal stump after laparoscopic hysterectomy.

Regarding the relationship between vaginal microecological imbalance and cellular immune inflammatory response, Th1 and Th2 immune cells play important regulatory and auxiliary roles in the body’s resistance to viruses, bacteria, and other pathogens (17). The levels of inflammatory factors produced (IL-6, IL-11, IL-2, and IL-21) are positively correlated with the degree of the inflammatory response, and these levels can be used to assess the degree of inflammation in the body (18). In our study, 3 days after therapy, the levels of CRP, IL-6, IL-11, IL-21, and IL-2 declined in both groups, and their levels in the observation group were lower than those in the control group. These results suggest that PRP gel may beneficially improve vaginal microecology in patients undergoing laparoscopic hysterectomy, potentially by influencing the inflammatory response. However, we cannot definitively conclude that PRP gel regulates the inflammatory response to achieve this effect based solely on the observed changes in inflammatory factor levels. Further research is needed to clarify the specific mechanisms. Xu et al. (19) suggested that PRP improves skin wound healing by modulating inflammation, which provides some indirect support for our hypothesis.

Toll-like receptor 4 (TLR4) is a transmembrane protein that can recognize various types of pathogen-associated molecular patterns (such as lipopolysaccharide, sodium urate crystals, and viral double-stranded RNA) and cause inflammatory immune responses in the body (20). The complement system is an important part of the innate immune system, which plays an important role in resisting the invasion of pathogenic microorganisms and in immune regulation (21). However, excessive activation of the complement system can produce a variety of inflammatory mediators to promote the development of inflammation (22). MBL is a type of agglutinin produced by the liver and monocytes that can activate the complement system (23). In blood circulation, MBL can activate MBL-related serine protease to form complement C3 invertase, and then initiate complement activation after cleavage into complement C3. Increased levels of MBL can lead to over-activation of the complement system and further promote the progression of inflammation (24). In our study, 3 days after therapy, TLR4 and MBL levels decreased in both groups, and their levels in the observation group were lower than those in the control group. These data suggest that PRP gel may improve vaginal microecology in patients undergoing laparoscopic hysterectomy by influencing TLR4 and MBL levels. However, as noted above, we cannot draw firm conclusions about the specific mechanisms based on these data alone. Liu et al. (25) reported that PRP alleviated endometritis induced by lipopolysaccharide in mice by suppressing the TLR4/NF-κB signaling pathway, which offers some related insights but does not directly confirm our findings.

### Clinical significance

The positive effects of PRP gel observed in our study have significant clinical implications. Laparoscopic hysterectomy is a common surgical procedure, and poor healing of the vaginal stump can lead to serious complications such as bleeding, infection, and even abdominal infection, which can significantly affect patients’ quality of life and increase healthcare costs. Our results show that PRP gel can effectively reduce the incidence of these complications, improve the healing quality of the vaginal stump, and enhance the overall vaginal microecological environment.

### Limitations

Our study has several limitations. First, there is a lack of exploration regarding causal pathways. Although we measured cytokine and immune regulator levels, the precise causal relationships, such as how PRP growth factors specifically influence TLR4 and MBL, were not fully investigated. This limits our in-depth understanding of the exact molecular mechanisms underlying the beneficial effects of PRP gel. Further research, including *in vitro* experiments and more detailed *in vivo* studies, is needed to elucidate these causal relationships and provide a more comprehensive understanding of the effects of PRP gel on the vaginal microenvironment. Second, important confounding factors were not accounted for in our research. We did not report on the use of antibiotics, perioperative medications, surgeon variability, or postoperative care. These factors could potentially influence the study outcomes, and their absence in our analysis may introduce bias and affect the validity of our results. Third, our study had a relatively small sample size and was conducted in a single center. This may restrict the generalizability of our findings. Larger-scale, multi-center studies are required to confirm the results and enhance the external validity of the study. Fourth, we did not perform microbiome sequencing. Given that the vaginal microenvironment is closely related to the vaginal microbiome, the lack of this analysis may prevent us from fully understanding the impact of PRP gel on the vaginal ecosystem. In addition, the scoring methods used in our study may be somewhat subjective, and there is a potential for examiner bias. This could affect the accuracy and reliability of our results. Finally, the follow-up period in our study was relatively short. A longer follow-up would be beneficial to assess the long-term effects of PRP gel on vaginal microecology and vaginal stump healing, as well as to detect any potential late-onset complications.

## Conclusion

Our study provides evidence that PRP gel positively affects vaginal microecology and promotes the healing of the vaginal stump in patients undergoing laparoscopic hysterectomy. The observed changes in relevant indicators suggest that PRP gel may be a promising therapeutic option. However, further research is needed to fully understand the underlying mechanisms, confirm the long-term effects, account for confounding factors, and improve the study design to enhance the generalizability and reliability of the findings.

## Data Availability

The datasets presented in this study can be found in online repositories. The names of the repository/repositories and accession number(s) can be found in the article/supplementary material.

## References

[1] Lyons TL. Laparoscopic supracervical hysterectomy. Obstet Gynecol Clin N Am. (2000) 27:441–50. ix 10.1016/s0889-8545(00)80034-0 10857133

[2] Chen S Zheng Q Zhang L Chen L Wang J. Effect of vaginal microecological alterations on female pelvic organ prolapse. Int Urogynecol J. (2024) 35:881–91. doi: 10.1007/s00192-024-05759-7, 38488886 PMC11052768

[3] Yang Y Yang H Ji J Zhao Y He Y Wu J. Predictive value of abdominal wall scar score for pelvic floor function rehabilitation, vaginal microecology and complications after cesarean section. PeerJ. (2023) 11:e16012. doi: 10.7717/peerj.16012, 37727692 PMC10506580

[4] Dohan Ehrenfest DM Rasmusson L Albrektsson T. Classification of platelet concentrates: from pure platelet-rich plasma (P-PRP) to leucocyte- and platelet-rich fibrin (L-PRF). Trends Biotechnol. (2009) 27:158–67. doi: 10.1016/j.tibtech.2008.11.009 19187989

[5] Everts PA Lana JF Alexander RW Dallo I Kon E Ambach MA . Profound properties of protein-rich, platelet-rich plasma matrices as novel, multi-purpose biological platforms in tissue repair, regeneration, and wound healing. Int J Mol Sci. (2024) 25:7914. doi: 10.3390/ijms25147914, 39063156 PMC11277244

[6] Chellini F Tani A Zecchi-Orlandini S Sassoli C. Influence of platelet-rich and platelet-poor plasma on endogenous mechanisms of skeletal muscle repair/regeneration. Int J Mol Sci. (2019) 20:683. doi: 10.3390/ijms20030683, 30764506 PMC6387315

[7] Rodríguez-Eguren A Bueno-Fernandez C Gómez-Álvarez M Francés-Herrero E Pellicer A Bellver J . Evolution of biotechnological advances and regenerative therapies for endometrial disorders: a systematic review. Hum Reprod Update. (2024) 30:584–613. doi: 10.1093/humupd/dmae013 38796750 PMC11369227

[8] Sharara FI Lelea LL Rahman S Klebanoff JS Moawad GN. A narrative review of platelet-rich plasma (PRP) in reproductive medicine. J Assist Reprod Genet. (2021) 38:1003–12. doi: 10.1007/s10815-021-02146-9, 33723748 PMC8190208

[9] Mouanness M Ali-Bynom S Jackman J Seckin S Merhi Z. Use of intra-uterine injection of platelet-rich plasma (PRP) for endometrial receptivity and thickness: a literature review of the mechanisms of action. Reprod Sci. (2021) 28:1659–70. doi: 10.1007/s43032-021-00579-2, 33886116

[10] Chrysanthopoulou EL Pergialiotis V Perrea D Κourkoulis S Verikokos C Doumouchtsis SK. Platelet rich plasma as a minimally invasive approach to uterine prolapse. Med Hypotheses. (2017) 104:97–100. doi: 10.1016/j.mehy.2017.05.018, 28673602

[11] Lin Y Qi J Sun Y. Platelet-rich plasma as a potential new strategy in the endometrium treatment in assisted reproductive technology. Front Endocrinol. (2021) 12:707584. doi: 10.3389/fendo.2021.707584, 34733236 PMC8558624

[12] Lv X Ding B Xu JY Shen Y. Effect of modified radical laparoscopic hysterectomy versus open radical hysterectomy on short-term clinical outcomes in early-stage cervical cancer: a single-center, prospective, randomized controlled trial. World J Surg Oncol. (2023) 21:167. doi: 10.1186/s12957-023-03044-3, 37270549 PMC10239128

[13] Qi J Li X Cao Y Long Y Lai J Yao Y . Locationally activated PRP via an injectable dual-network hydrogel for endometrial regeneration. Biomaterials. (2024) 309:122615. doi: 10.1016/j.biomaterials.2024.122615, 38759486

[14] Mao L Wang XX Sun Y Yang M Chen X Cui L . Platelet-rich fibrin improves repair and regeneration of damaged endometrium in rats. Front Endocrinol. (2023) 14:1154958. doi: 10.3389/fendo.2023.1154958, 37614713 PMC10443704

[15] Park HS Ulin M Cetin E. Ovarian rejuvenation using platelet-rich plasma: a promising option for women in early menopause to have a baby. Reprod Sci. (2020) 27:1983–4. doi: 10.1007/s43032-020-00315-2, 32935255

[16] Garavelas A Mallis P Michalopoulos E Nikitos E. Clinical benefit of autologous platelet-rich plasma infusion in ovarian function rejuvenation: evidence from a before-after prospective pilot study. Medicines. (2023) 10:19. doi: 10.3390/medicines10030019, 36976308 PMC10056078

[17] Bi H Zhang D Xiao B. Association between human papillomavirus infection and common sexually transmitted infections, and the clinical significance of different Mycoplasma subtypes. Front Cell Infect Microbiol. (2023) 13:1145215. doi: 10.3389/fcimb.2023.1145215 37009504 PMC10061082

[18] Rabkin SW. Nitric oxide and peroxynitrite induce gene expression of interleukin receptors increasing IL-21, IL-7, IL-1 and oncostatin M in cardiomyocytes. Life Sci. (2010) 86:45–51. doi: 10.1016/j.lfs.2009.11.002, 19913562

[19] Xu P Wu Y Zhou L Yang Z Zhang X Hu X . *Platelet-rich plasma accelerates skin wound healing by promoting re-epithelialization.* Burns. Trauma. (2020) 8:tkaa028. doi: 10.1093/burnst/tkaa028, 32821743 PMC7427034

[20] Jackson Hoffman BA Pumford EA Enueme AI Fetah KL Friedl OM Kasko AM. Engineered macromolecular toll-like receptor agents and assemblies. Trends Biotechnol. (2023) 41:1139–54. doi: 10.1016/j.tibtech.2023.03.008, 37068999

[21] Elieh Ali Komi D Shafaghat F Kovanen PT Meri S. Mast cells and complement system: ancient interactions between components of innate immunity. Allergy. (2020) 75:2818–28. doi: 10.1111/all.14413 32446274

[22] West EE Kemper C. Complosome - the intracellular complement system. Nat Rev Nephrol. (2023) 19:426–39. doi: 10.1038/s41581-023-00704-1, 37055581 PMC10100629

[23] Dørflinger GH Høyem PH Laugesen E Østergaard JA Funck KL Steffensen R . High MBL-expressing genotypes are associated with deterioration in renal function in type 2 diabetes. Front Immunol. (2022) 13:1080388. doi: 10.3389/fimmu.2022.1080388, 36618347 PMC9816478

[24] Beinrohr L Dobó J Závodszky P Gál P. C1, MBL-MASPs and C1-inhibitor: novel approaches for targeting complement-mediated inflammation. Trends Mol Med. (2008) 14:511–21. doi: 10.1016/j.molmed.2008.09.009, 18977695

[25] Liu X Wang Y Wen X Hao C Ma J Yan L. Platelet rich plasma alleviates endometritis induced by lipopolysaccharide in mice via inhibiting TLR4/NF-κB signaling pathway. Am J Reprod Immunol. (2024) 91:e13833. doi: 10.1111/aji.13833, 38467595

